# Variability of insulin sensitivity during the first 4 days of critical illness: implications for tight glycemic control

**DOI:** 10.1186/2110-5820-2-17

**Published:** 2012-06-15

**Authors:** Christopher G Pretty, Aaron J Le Compte, J Geoffrey Chase, Geoffrey M Shaw, Jean-Charles Preiser, Sophie Penning, Thomas Desaive

**Affiliations:** 1 Department of Mechanical Eng, Centre for Bio-Engineering, University of Canterbury, Private Bag 4800, Christchurch, 8054, New Zealand; 2 Department of Intensive Care, Christchurch Hospital, Christchurch, 8054, New Zealand; 3 Department of Intensive Care, CUB Hospital Erasme, Free University of Brussels, Route de Lennik 808, Brussels, 1070, Belgium; 4 Cardiovascular Research University of Liege, Liege, Belgium

**Keywords:** Critical care, Hyperglycemia, Insulin resistance, Mathematical model, Algorithms

## Abstract

**Background:**

Effective tight glycemic control (TGC) can improve outcomes in critical care patients, but it is difficult to achieve consistently. Insulin sensitivity defines the metabolic balance between insulin concentration and insulin-mediated glucose disposal. Hence, variability of insulin sensitivity can cause variable glycemia. This study quantifies and compares the daily evolution of insulin sensitivity level and variability for critical care patients receiving TGC.

**Methods:**

This is a retrospective analysis of data from the SPRINT TGC study involving patients admitted to a mixed medical-surgical ICU between August 2005 and May 2007. Only patients who commenced TGC within 12 hours of ICU admission and spent at least 24 hours on the SPRINT protocol were included (N = 164). Model-based insulin sensitivity (*SI*) was identified each hour. Absolute level and hour-to-hour percent changes in *SI* were assessed on cohort and per-patient bases. Levels and variability of *SI* were compared over time on 24-hour and 6-hour timescales for the first 4 days of ICU stay.

**Results:**

Cohort and per-patient median *SI* levels increased by 34% and 33% (*p* < 0.001) between days 1 and 2 of ICU stay. Concomitantly, cohort and per-patient *SI* variability decreased by 32% and 36% (*p* < 0.001). For 72% of the cohort, median *SI* on day 2 was higher than on day 1. The day 1–2 results are the only clear, statistically significant trends across both analyses. Analysis of the first 24 hours using 6-hour blocks of *SI* data showed that most of the improvement in insulin sensitivity level and variability seen between days 1 and 2 occurred during the first 12–18 hours of day 1.

**Conclusions:**

Critically ill patients have significantly lower and more variable insulin sensitivity on day 1 than later in their ICU stay and particularly during the first 12 hours. This rapid improvement is likely due to the decline of counter-regulatory hormones as the acute phase of critical illness progresses. Clinically, these results suggest that while using TGC protocols with patients during their first few days of ICU stay, extra care should be afforded. Increased measurement frequency, higher target glycemic bands, conservative insulin dosing, and modulation of carbohydrate nutrition should be considered to minimize safely the outcome glycemic variability and reduce the risk of hypoglycemia.

## Background

Safe, effective tight glycemic control (TGC) of critically ill patients can improve outcomes [1-4], but it is difficult to achieve consistently [5-7]. Glycemic level and variability in TGC are a function of variability in insulin sensitivity, potentially resulting from the level and evolution of the stress response [8], and are independently associated with mortality [9-12].

Insulin sensitivity defines the metabolic balance between insulin concentration and glucose disposal. Insulin-mediated glucose disposal is a dominant pathway to reduce and control glycemia in critically ill patients. For a fixed insulin concentration, a given percentage change of insulin sensitivity results in a proportional change to glucose disposal and thus glycemic level, all else equal.

Understanding the variability of insulin sensitivity, over hours and days, is important for safely and effectively managing glycemic levels with exogenous insulin. Several patient- and treatment-related factors influence insulin sensitivity. Some of the influential and predictable factors (drug therapies and existing patient conditions) are taken into account when developing therapeutic algorithms for insulin treatment.

The objective of this study was to examine the evolution of insulin sensitivity level and variability over the first 4 days of intensive care unit (ICU) stay using data from the SPRINT TGC study [1]. Analyses were performed on two separate timescales, using 24-hour and 6-hour blocks of data. The impact of this insulin sensitivity evolution on glycemia in the context of TGC protocols is considered.

## Methods

### Patients

This study is a retrospective analysis of patient data (N = 164 patients, 12,067 hours) from the SPRINT clinical practise change in the Christchurch Hospital ICU [1]. All patients admitted between August 2005 and May 2007 were included where the SPRINT TGC protocol was commenced within 12 hours of ICU admission and continued for at least 24 hours. All patients were treated per protocol, with no specific exclusions. Table 1 presents a summary of cohort details. 

**Table 1:** Summary details of the study subjects

**N**	**164**
Age (yr)	65 [56–74]
Gender (M/F)	102/62
APACHE II score	19 [16–25]
APACHE II ROD (%)	32 [17–52]
Operative/nonoperative	66/98
Hospital mortality	25%
ICU mortality	18%
ICU length of stay (hr)	142 [70–308]
Diabetic history: type I/type II	10/22

Data are presented as median [interquartile range] where appropriate.

The Christchurch Hospital ICU is a 15-bed, closed, mixed medical-surgical unit led by intensive care specialists in a tertiary affiliated teaching hospital. Glycemic control data were collected from handwritten daily ICU charts and entered into a spreadsheet database. The Upper South Regional Ethics Committee, New Zealand, granted approval for the audit, analysis, and publication of this data.

### The SPRINT protocol

The SPRINT protocol (SPecialised Relative Insulin Nutrition Tables) is a simple, lookup-table system derived from a model-based controller that modulates both insulin and nutritional inputs. The protocol titrates insulin doses and nutrition rates to estimated patient-specific insulin sensitivity for tight glycemic control in the range 4.0–6.1 mmol/L BG range [1,13,14]. SPRINT has been the standard of care in the Christchurch ICU since August 2005. The requirement for the patients in this study to be on the SPRINT protocol ensured that they had regular and accurate records of blood glucose levels, insulin administered, and nutrition given.

The entry criterion for the SPRINT protocol was two BG measurements >8 mmol/L during normal patient monitoring, or at the discretion of the clinician. Once on the protocol, BG was measured 1- to 2-hourly, with a median measurement interval for this cohort of 1.5 hours. BG measurements were taken by nursing staff using the Arkray Super-Glucocard II glucometer (Arkray Inc., Japan). Blood samples tested were typically arterial, although when an arterial line was not present, capillary blood was used. Additional File 1 contains a more detailed description of SPRINT and specific, unique differences to other protocols.

### Model-based insulin sensitivity

**Figure 1:**
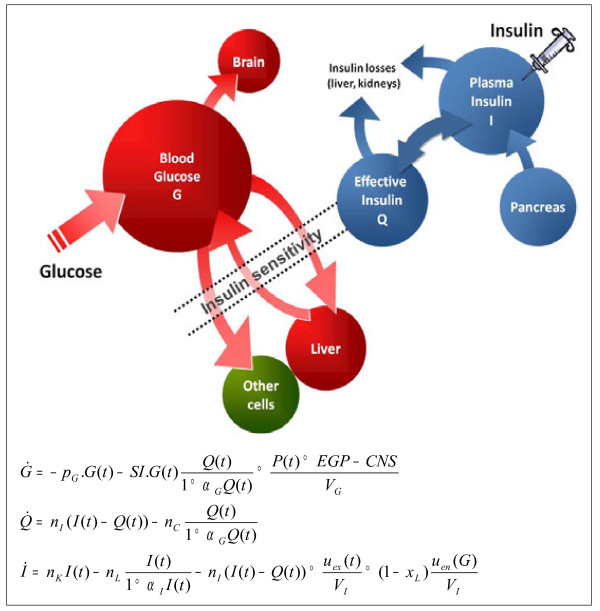
Schematic illustration of the glucose-insulin system model used in this analysis.

Model-based methods provide a means of determining physiological parameters that either cannot be measured directly or are impractical to measure with the required frequency. In this study, model-based insulin sensitivity (*SI*) was identified using an integral method [15] with a validated glucose-insulin system model developed for critical care patients [16,17]. The glucose-insulin system model is illustrated schematically in Figure 1 and presented in greater detail in Additional File 2. 

The *SI* parameter represents “whole-body” insulin sensitivity. The parameter defines the glycemic response to exogenous insulin and nutrition, capturing the relative net effect of altered endogenous glucose production, peripheral and hepatic insulin mediated glucose uptake, and endogenous insulin secretion. However, this time-varying insulin sensitivity parameter has been shown to correlate very well (r > 0.9) with the “gold standard” euglycemic clamp [17] and has been used to guide model-based TGC in several studies [18-20].

A value of *SI* was identified every hour [15] for each patient using clinical data and the model implemented in MATLAB (2011a, Mathworks, Natick, MA). When the BG measurement interval was greater than 1 hour, linearly interpolated values were used for identification. Variability of insulin sensitivity was calculated as the hour-to-hour percentage change in *SI* (Δ%*SI*), defined below:

(1) $$\Delta \%{SI}_{k}=100\times \frac{\left({SI}_{k+1}-{SI}_{k}\right)}{{SI}_{k}}$$

Use of percentage change in *SI*, rather than absolute change, normalizes the metric so that patients with very different absolute levels of *SI* can be compared fairly. Equally, for a fixed insulin concentration, a given percentage change in insulin sensitivity results in a proportional change to glucose disposal and thus glycemic level, all else equal.

### Analyses

*SI* level and variability are analyzed on overall cohort and per-patient bases using two separate timescales. The evolution of *SI* over the first 4 days of ICU stay is analyzed in 24-hour blocks. Bagshaw [12] reported an association between hypoglycemia and variability during the first 24 hours of ICU stay and mortality. We therefore also analyzed the acute evolution of *SI* over the first day using 6-hour blocks.

Cohort analysis looks at the hourly values of *SI* and variability for the entire cohort grouped together and shows trends in the overall group behavior. To quantify per-patient variability, the interquartile range (IQR: 25^th^–75^th^ percentile) of Δ%*SI* is examined for each patient within each timescale. This metric captures the width of the variability distribution for each patient. Per-patient *SI* level is defined by the median value within each timescale.

The analyses are linked to time on the SPRINT protocol, rather than time in the ICU, to ensure sufficient insulin and nutrition data to accurately identify *SI* hourly [15]. Hence, day 1 comprises the first 24 hours of SPRINT. However, because patients were included only if they commenced SPRINT within 12 hours of ICU admission, a minimum of half of the day 1 results for each patient occur during their first 24 hours in the ICU. The median delay between admission and commencement of SPRINT for this cohort was 1.9 hours and 81% of the cohort was on SPRINT within 6 hours. When a patient was taken off the SPRINT protocol, their *SI* profile for the last day was included in the analysis only if it contained 6 hours or more of data.

*SI* levels and variability are non-Gaussian and thus compared using cumulative distribution functions (CDFs) and nonparametric statistics. Distributed data are generally compared using the Wilcoxon rank-sum test (Mann–Whitney *U* test), except for *SI* variability results. *SI* variability is compared using the Kolmogorov-Smirnov test, because it has more power to detect differences in the shape of distributions than the rank-sum test when median values are similar. *P* < 0.05 are considered statistically significant.

## Results

### Twenty-four hour analyses

#### *Insulin sensitivity level*

**Figure 2:**
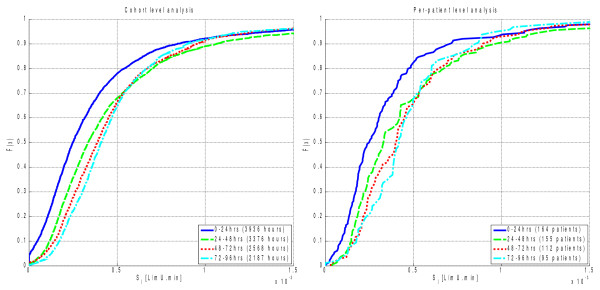
Insulin sensitivity level distributions by cohort (left) and per-patient median (right) using 24-hr blocks of data.

Figure 2 presents the cumulative distribution functions (CDFs) of hourly *SI* for each day by cohort (left panel) and median daily *SI* per-patient (right panel). Table 2 presents the increase in median insulin sensitivity and associated *p* values between successive days. Both per-patient and cohort analyses suggest that insulin sensitivity levels start low, but increase over time in the ICU. There is a particularly significant increase between days 1 and 2 (*p* < 0.001). On subsequent days the increase continues but to a lesser degree. Per-patient comparisons between days 2, 3, and 4 are not statistically significant.

**Table 2:** Increasing cohort and per-patient median insulin sensitivity over time (24-hr blocks)

**Level analysis**	**Cohort analysis**		**Per-patient analysis**	
	**% Increase at median**	***p *value**	**% Increase at median**	***p *value**
Days 1-2	34	<0.0001	33	0.0004
Days 2-3	16	<0.0001	21	0.2559
Days 3-4	6	0.0013	4	0.6306

*P* values calculated using Wilcoxon rank-sum test.

The results of Figure 2 and Table 2 are further reflected in Table 3, which shows that daily median insulin sensitivity increases for a large proportion of the cohort between days 1 and 2 with lesser proportions on subsequent days. Table 3 is a matrix where the value in a cell represents the proportion of patients for whom daily median insulin sensitivity is greater on the day of the associated column than the day of the associated row. For example, 72% of patients show an increase in median *SI* between days 1 and 2, and 54% when comparing days 2 and 3.

**Table 3:** Proportion of patients for whom median insulin sensitivity increases between the days indicated in the rows and columns

	**Day 2**	**Day 3**	**Day 4**
**Day 1**	0.72	0.74	0.71
**Day 2**		0.54	0.64
**Day 3**			0.53

#### *Insulin sensitivity variability*

**Figure 3:**
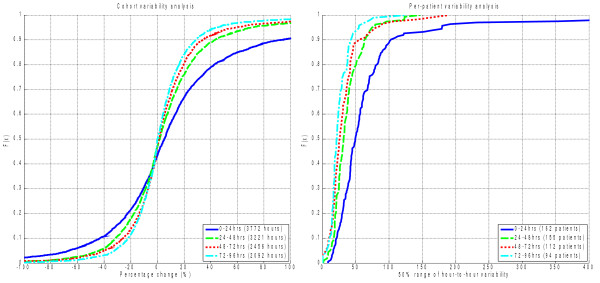
Insulin sensitivity variability distributions by cohort (hour-to-hour percentage change) and per-patient interquartile-range using 24 hr blocks of data.

*SI* variability decreases over time in the ICU, parallel to increases in absolute *SI* level. Figure 3 and Table 4 present the CDFs and tabulated results for cohort and per-patient analyses of the hour-to-hour percentage changes in *SI* (Δ%*SI*). The cohort aggregate distributions of Δ%*SI* by day are shown in the left panel of Figure 3. The right panel presents the CDFs for the per-patient IQRs by day.

**Table 4:** Reductions in the interquartile range (IQR) and median per-patient range of hour-to-hour percentage insulin sensitivity change over time

**Variability analysis**	**Cohort analysis**		**Per-patient analysis**	
	**% Reduction of IQR**	**p-value**	**% Decrease at median**	***p* value**
**Days 1-2**	32	<0.0001	36	<0.0001
**Days 2-3**	20	0.0028	18	0.0091
**Days 3-4**	14	0.0269	17	0.0369

*P* values calculated using Kolmogorov-Smirnov test for cohort comparisons and Wilcoxon rank-sum test for per-patient comparisons.

As with insulin sensitivity level, the largest increase in *SI* variability is between days 1 and 2. The decrease between days 2, 3, and 4 is statistically significant for both cohort and per-patient analyses, but the change is much less than over the first day and may not be clinically significant.

### Six-hour analyses

#### *Insulin sensitivity level*

**Figure 4:**
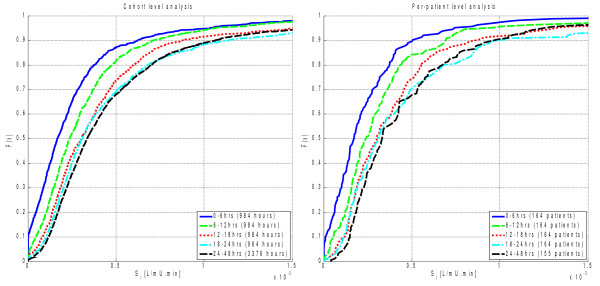
Insulin sensitivity level distributions by cohort (left) and per-patient median (right) using 6-hr blocks of data.

Figure 4 presents the distributions of cohort and per-patient insulin sensitivity over the first 24 hours in 6-hour blocks. Also shown for comparison is the day 2 distribution from Figure 1 (labeled 24–28 hours). It is evident that the insulin sensitivity level increases over the first day up to the level of the second day. Hence, the differences between day 1 and 2 seen in Figure 2 are a function of the low, but increasing, insulin sensitivity during the first 12–18 hours.

Table 5 lists the differences in median insulin sensitivity levels from the distributions shown in Figure 4. The increases in *SI* during the first 18 hours are large and statistically significant. Subsequent increases are unlikely to be clinically significant at less than 10%. Of particular interest is the comparison between 18–24 hours and day 2, which indicates that by 18 hours, the rapid increase in *SI* is largely complete.

**Table 5:** Increasing cohort and per-patient median insulin sensitivity over time (6-hr blocks)

**Level analysis**	**Cohort analysis**		**Per-patient analysis**	
	**% Increase at median**	***p *value**	**% Increase at median**	***p *value**
**Block 1–2 (0–6 vs. 6–12 hr)**	42	<0.0001	40	0.0007
**Block 2–3 (6–12 vs. 12–18 hr)**	28	<0.0001	26	0.0123
**Block 3–4 (12–18 vs. 18–24 hr)**	1	0.0335	3	0.4829
**Block 4–5 (18–24 vs. 24–48 hr)**	9	0.0452	7	0.3776

*P* values calculated using Wilcoxon rank-sum test.

Table 6 shows that during the first 18 hours, a large proportion of the patients have an increase of insulin sensitivity using the 6-hour timescale. After 18 hours, the proportion of patients with increasing *SI* is similar to that seen between days 2, 3, and 4 (Table 3) at slightly more than 50%.

**Table 6:** Proportion of patients for whom median insulin sensitivity increases between the blocks indicated in the rows and columns

	**6–12 hr**	**12–18 hr**	**18–24 hr**	**24–48 hr**
**0–6 hr**	0.74	0.78	0.77	0.79
**6–12 hr**		0.76	0.7	0.72
**12–18 hr**			0.55	0.64
**18–24 hr**				0.58

#### *Insulin sensitivity variability*

**Figure 5:**
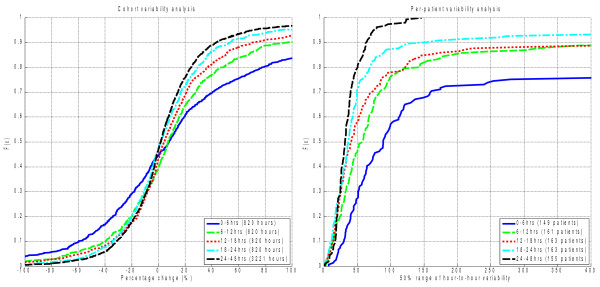
Insulin sensitivity variability distributions by cohort (hour-to-hour percentage change) and per-patient interquartile-range using 6-hr blocks of data.

As with absolute *SI* level, the majority of the decrease in *SI* variability occurred during the first 18 hours. Figure 5 shows the CDFs of the cohort and per-patient variability metrics. Table 7 shows that only the differences between 0–6 hours and 6–12 hours are statistically significant at the 5% level. The 6–12 vs. 12–18-hour comparison is close to statistical significance, with *p* < 0.07 for both cohort and per-patient analyses.

**Table 7:** Reductions of the interquartile range (IQR) and median per-patient range of hour-to-hour percentage insulin sensitivity change over time

**Variability analysis**	**Cohort analysis**		**Per-patient analysis**	
	**% Reduction of IQR**	***p *value**	**% Decrease at median**	***p *value**
**Block 1–2 (0–6 vs. 6–12 hr)**	40	0.0017	36	<0.0001
**Block 2–3 (6–12 vs. 12–18 hr)**	24	0.0628	28	0.0673
**Block 3–4 (12–18 vs. 18–24 hr)**	0	0.0931	9	0.1032
**Block 4–5 (18–24 vs. 24–48 hr)**	18	0.1682	14	0.1075

*P* values calculated using Kolmogorov-Smirnov test for cohort comparisons and Wilcoxon rank-sum test for per-patient comparisons.

## Discussion

### Insulin sensitivity variability

Both cohort and per-patient results suggest that critically ill patients have significantly lower and more variable insulin sensitivity on day 1 than later in their ICU stay. Further analysis shows that this day 1 result is primarily influenced by the first 12–18 hours of ICU stay. Over this time, rapid improvements in insulin sensitivity level and variability occur so that there is no statistically significant difference between 18–24 hours and day 2. From day 2 onwards, changes in *SI* level and variability are not as large and of limited clinical and statistical significance.

Within the analyses, there are some differences in significance between cohort and per-patient results for comparisons after day 2. The overall findings noted in the preceding paragraph are the only clear, consistent trends across both analyses.

The counter-regulatory hormones: cortisol, glucagon, the catecholamines, as well as growth hormone are significantly elevated almost immediately after critical-insult, but decline rapidly over the first 12–48 hours [21-24]. These hormones are known to cause increased hepatic glucose production, inhibition of insulin release, and peripheral insulin resistance [22], all of which cause a decrease in the model-based *SI* metric used in this study. Hence, the low but rapidly increasing insulin sensitivity seen during the first 12–18 hours of ICU stay is likely due to the acute counter-regulatory response to critical illness.

Time in this study was referenced from the commencement of SPRINT, rather than ICU admission. However, the difference between admission time and commencing SPRINT was generally very short, with a median delay for this cohort of 1.9 hours. Within 6 hours of admission, 81% of the cohort had commenced SPRINT. Hence, these results are applicable to the first few hours and days of ICU stay.

### The insulin sensitivity parameter

The model-based parameter used in this study represents a whole-body insulin sensitivity capturing overall metabolic response to exogenous insulin. *SI* captures the relative net effect of altered hepatic glucose production, peripheral and hepatic insulin-mediated glucose uptake, and endogenous insulin secretion. All of these effects are altered significantly in critical illness due to the stress response [25-27]. Hence, the metabolic balance that this parameter represents is an important consideration in TGC, because it determines a body’s glycemic response to exogenous insulin and nutrition.

As an identified parameter, *SI* contains unmodeled physiological effects and measurement device noise. However, Lotz et al. [17] indicated that this form of insulin sensitivity correlated very well (r > 0.9) with the “gold standard” euglycemic clamp and its change in a lifestyle intervention study on 73 normoglycemic healthy and obese subjects (146 clamp procedures before/after intervention). In the critical care setting, a similar version of the model and *SI* parameter has been cross-validated against independent, matched patient data from a single center of the Glucontrol randomized, clinical trial [28].

The analytic inaccuracy of bedside glucometers or any other sensor used to gather BG measurements influence individual values of *SI*. However, this study examines distributions of *SI* consisting of thousands of values identified from a wide range of BG values, thus both the random and bias components of error cancel out within each distribution. This effect was confirmed by Monte Carlo analysis (results not shown) using an error model for the glucometer derived from data supplied by the manufacturer [29].

### Implications for tight glycemic control

With low and variable insulin sensitivity, glycemic levels may appear unresponsive and/or difficult to control effectively with exogenous insulin. This situation may provoke larger insulin doses from many protocols that have no explicit upper limits on insulin dose [6,30-32]. High levels of circulating insulin coupled with the observed variability in insulin sensitivity result in increased glycemic variability and an increased risk of hypoglycemia during the first 24 hours of ICU stay.

Not only does glycemic variability pose a risk through hypoglycemia, it also is detrimental in its own right. Several studies [9-11,33] have shown that glycemic variability is independently associated with mortality in critically ill patients. More specifically, Bagshaw [12] showed that hypoglycemia and variability within the first 24 hours of ICU stay are each associated with increased mortality. In vitro, high glycemic variability was shown to increase oxidative stress [34] and apoptosis [35], thereby suggesting a rationale to explain the clinical association with poor outcome.

Evidence from other studies [10,12] indicates an association between hypoglycemia, glycemic variability, and mortality. However, the question remains: Is low and variable glycemia the cause of increased morbidity and mortality? Or is it just a symptom in very ill patients? Until this question can be answered conclusively, it is perhaps best to formulate TGC protocols not to exacerbate the situation, which requires the ability to differentiate more and less metabolically variable patients.

Another significant finding in this study is the range of variability seen across patients, as well as over time (Figures 3 and 5). Less variable patients, if identified, may be treated more aggressively with insulin without compromising glycemic variability. Hence, model-based methods have been mooted as a means of better managing this inter- and intra-patient variability [30,36].

### Limitations

Only patients on the SPRINT TGC protocol were considered for this analysis as they had sufficient data density to identify *SI* hourly. Patients were put on the SPRINT protocol because they were hyperglycemic and thus were likely to be biased towards lower insulin sensitivity compared with other ICU patients. However, in the context of investigating the implications of *SI* variability on TGC, this cohort is appropriate.

Another limitation is the use of a model-based insulin sensitivity parameter, as it is not measured directly and may be influenced by modelling errors or un-modelled effects. As an identified parameter, *SI* contains unmodeled physiological effects and measurement device noise. However, as noted previously, this form of *SI* has been shown to correlate very well with the “gold standard” euglycemic clamp [17,37] and has been shown to be an independent marker of metabolic condition [28]. Finally, this method of analysis is robust to BG sensor error.

A further limitation is the relatively small cohort size available for analysis. The demands of manually transcribing written clinical data into electronic form and the specific inclusion criteria have restricted the number of patients for whom complete glycemic control data are currently available for analysis. The size of this cohort has precluded subgroup analyses, such as diabetic and cardiovascular surgery patients, because these subgroups only contain 20–40 patients. With relatively few patients, the subgroup analyses fail to demonstrate statistical significance, despite effect sizes and trends very similar to that seen in this overall analysis. Thus, these comparisons will be completed in the future, when more patient data become available.

The findings of this study should be equally valid in other ICUs where attention to TGC and blood glucose measurement frequency may be a lower priority. Although the data density might not be present to allow such units to explicitly identify *SI* hourly, these results indicate that patients will still have lower and more variable insulin sensitivity on day 1 than later in their ICU stay. Thus, suggestions of higher glycemic targets, conservative insulin dosing, and modulation of carbohydrate nutrition are especially pertinent.

Without the ability to identify patient-specific metabolic states, a protocol should be less aggressive over the first few days, and particularly the first 24 hours, to minimize variability. It may be important for protocols to consider higher glycemic targets on the first days of ICU stay (compared with later days) to ensure safety. Perhaps a glycemic target similar to the current guidelines of 7.8-11 mmol/L [38-40] is most appropriate for the first 24 hours with the target range, reducing over days 2 and 3 to more normoglycemic levels as *SI* level and variability improve.

Greater blood glucose measurement frequency and conservative insulin dosing can mitigate the impact of *SI* variability on risk [41] and also should be considered for the first few days of stay. Modulation of carbohydrate nutrition, within limits [42], can reduce the need for exogenous insulin to better manage glycemia [43].

## Conclusions

The results of this study indicate that critically ill patients have significantly lower and more variable insulin sensitivity on day 1 than later in their ICU stay, particularly during the first 12–18 hours. This effect is likely due to the acute counter-regulatory response to critical illness. Greater variability with lower *SI* early in a patient’s stay greatly increases the insulin required, potential glucose flux due to variation in *SI*, and thus the risk of greater glycemic variability and hypoglycemia. Both glycemic variability and hypoglycemia have been associated with poor outcomes in the ICU.

Clinically, these results suggest that TGC patients require greater care over the first few days of ICU stay to minimize safely the outcome glycemic variability. It may be important for protocols to consider higher glycemic targets on the first days of ICU stay to ensure safety. Equally, greater measurement frequency, conservative insulin dosing, and modulation of carbohydrate nutrition can mitigate the impact of variability on risk and should be considered for the first few days of stay.

## Abbreviations

ICU: Intensive care unit; SPRINT: Specialized relative insulin and nutrition titration; TGC: Tight glycemic control; *SI*: Insulin sensitivity metric (model-based); Δ%*SI*: Hour-to-hour percentage changes in insulin sensitivity; CDF: Cumulative distribution function; IQR: Interquartile range; APACHE: Acute physiology and chronic health evaluation; KS: Kolmogorov-Smirnov (test).

## Competing interests

The authors declare that they have no competing interests.

## Authors’ contributions

JGC, GS, and ALC conceived and developed the SPRINT protocol. GS implemented the protocol with staff at Christchurch Hospital. CGP, ALC, JGC, GS, TD, J-CP, and SP assisted with the data analysis, idea generation, some (or all) data collection, and/or the analysis and interpretation of the data and/or statistical analysis. CGP, JGC, and ALC drafted the manuscript primarily, although all of the authors made contributions. All authors read and approved the final manuscript.

## Supplementary Material

Additional file 1 Detailed description of the SPRINT protocol, listing unique features and differences to other TGC protocols. Click here for file

Additional file 2 **Detailed description of the glucose-insulin system model and the *SI* parameter [**[19,43-74]**].** Click here for file
