# Supplementary material for: Variability of insulin sensitivity during the first 4 days of critical illness: implications for tight glycemic control
Source: Ann Intensive Care. 2012 Jun 15;2:17. doi: 10.1186/2110-5820-2-17 (PMC3464183; doi:10.1186/2110-5820-2-17)
Supplement: Additional file 1 — Detailed description of the SPRINT protocol, listing unique features and differences to other TGC protocols. [file 2110-5820-2-17-S1.docx]

**APPENDIX 1**: **SPRINT Protocol Details and Differences to other TGC Protocols**

This Appendix is designed to present the SPRINT protocol referenced in this paper, including the features that make it unique compared with other TGC protocols. A separate set of references (from the main article) are used and given at the end of this Appendix.

**A1-1: The Protocol**: The entry criterion for the SPRINT protocol was a blood glucose measurement greater than 8 mmol/L on two occasions during standard patient monitoring. Patients were sometimes put on SPRINT at the discretion of the clinician if the blood glucose levels were consistently greater than 7 mmol/L in severe critical illness.

Hourly blood glucose measurements were used to ensure tight control [41]. Two-hourly measurements were used when the patient was stable, defined as 3 consecutive 1-hourly measurements in the 4.0-6.0 mmol/L band [41, 14], or when an arterial line was no longer present. SPRINT was stopped when the patient was adequately self regulating, defined as 6 or more hours (three 2-hourly measurements) in the 4.0-6.0 mmol/L band with over 80% of goal feed rate and a maximum of 2U/hour of insulin [41, 14]. All measurements were made with bedside glucometers (Arkray Super-Glucocard II, ArkrayInc. Japan) with typical errors in the range 7-12%. Hypoglycaemia was defined as BG < 4.0 mmol/l (mild) and BG < 2.2 mmol/L (severe).

Total insulin prescribed by SPRINT was limited to 6U/hr to minimise saturation and the administration of ineffective insulin [44-47]. Insulin was given predominantly in bolus form for safety, avoiding infusions being left on at levels inappropriate for evolving patient condition. Occasionally, clinicians prescribed a background insulin infusion rate of 0.5–1.0 U/hr, primarily for patients known to have Type II diabetes, and the insulin bolus recommendations from SPRINT were added to this rate. A background rate of 0.5–1.0 U/hr could also be mandated in patients with Type I diabetes.

Goal enteral nutrition rates were approximately 25 kcal/kg/day of RESOURCE Diabetic (Novartis Medical Nutrition, Minneapolis, MN) or Glucerna (Abbott Laboratories, Chicago, IL) with 34-36% of calories from carbohydrates. Maximum and minimum nutrition rates were 7.5 to 25 kcal/kg/day with 2.7 to 9 kcal/kg/day from carbohydrate. Thus, an 80kg male would receive maximum of 2000 kcal/day and a minimum of 600 kcal/day, with 216-640 kcal/day from carbohydrate, exceeding the minimum level below which there is an increased risk of bloodstream infections [48]. These guidelines are detailed in [20] and are effectively equivalent to the ACCP/SCCM guidelines [49].

Figures A1-1 - A1-4 show the SPRINT wheels that define the protocol, as well as stopping and measurement frequency criteria flowcharts. These wheels were used each 1-2 hours to determine the insulin and nutrition interventions. As noted, interventions are based on response to both inputs, effectively titrating on patient-specific insulin sensitivity displayed by that response.

**A1-2: Specific Features and Differences of SPRINT**: The SPRINT approach to TGC was unique in several areas from almost all other protocols. Specific differences are outlined below in terms of broad areas wherein differences lie:

- **Nutrition and Carbohydrate Intake**:
  - SPRINT directly controlled both insulin and nutrition where virtually all other protocols do not explicitly account for nutrition or carbohydrate intake. Hence, they dose insulin “blind” to carbohydrate intake
  - SPRINT specified the specific enteral and other nutritional sources, using low carbohydrate formulations with only 35-40% calories from carbohydrate. As a result, SPRINT had lower carbohydrate loading but within the guidelines for improved mortality reported by [42].
- **Interventions and TGC Approach**: The following issues defined the salient differences between SPRINT and other protocols in terms of how SPRINT was implemented, its limits on interventions, and how it titrates interventions on patient-specific insulin sensitivity (1/insulin resistance):
  - SPRINT controls both insulin and nutrition in concert and within safe ranges.
  - SPRINT specified insulin and nutrition interventions based on patient-specific insulin sensitivity. Thus, these interventions are not based on glycaemic response, as in almost all other algorithms, but on glycaemic response to patient-specific interventions.
    - Insulin sensitivity captures the patient-specific response to the inflammatory and counter-regulated critically ill patient state. Hence, it makes sense to titrate on estimates of this value to adjust interventions.
    - This approach cannot be taken without direct knowledge of the carbohydrate dosing given, making SPRINT unique.
  - SPRINT had a relatively quite low maximum insulin limit of 6U/hour to avoid saturation effects and also the risk of hypoglycaemia. Most published protocols utilise much higher maximum rates of insulin dosing, with some allowing up to 10-30 units or more per hour (e.g. [50, 32]).
    - Uniquely, SPRINT gives insulin in bolus form for safety as an infusion can be left running when not desirable and a bolus cannot.
  - SPRINT stops insulin if a blood glucose drop is “large” defined as 1.5 mmol/L in 1-2 hours with a current BG measurement less than 7 mmol/L (see Figure A1-2), which is above the target band of 4.0-6.0 mmol/L. In contrast, many published protocols do not stop insulin unless hypoglycaemia occurs and/or reduce the rate when insulin had passed below the glycaemic target band [32, 51], at which point it is often too late to avoid severe hypoglycaemic events.
  - SPRINT modulates its interventions very slowly with over 90% of possible outcomes requiring a change of only 1U/hour of insulin and/or 10% change in feed rate. This relatively slow approach to titrating blood glucose minimised rapid changes and the potential for hypoglycaemia.
  - SPRINT measured and intervened 1-2 hourly with no 3-4 hourly or longer intervals, ensuring tighter potential control over highly dynamic patients compared to most protocols that allow 4-hourly measurement, which for highly dynamic patients can result in loss of control as patient condition evolves.
  - SPRINT measured 2-hourly only in stable patients. Stable patients were defined as in the target band of 4-6 mmol/L and had relatively high insulin sensitivity (3U/hour or less of insulin and 60% or more of patient-specific goal feed rate).
    - Higher insulin sensitivity and correspondingly lower insulin usage implies a patient in relatively better condition and one with less change in glycaemia due to dynamic changes in patient condition.
    - This is a unique definition of stability compared to other protocols, and a more rigorous or harder definition to meet than merely being well controlled or in a target band.

Overall, SPRINT focused on patient-specific response to insulin and nutrition interventions, or effective insulin sensitivity to titrate interventions and define stability. As a result, with more frequent measurement, it was able to obtain tighter control across the range of dynamics and variability seen in these patients.

The following glycaemic outcomes were observed that are relevant to the tightness of control and/or unique to this protocol:

- SPRINT had higher times in all target glycaemic bands than other protocols [51] and thus tighter (less variable) control. In particular, the 97^th^ percentile patient over 50% of all blood glucose values in a 4.0-7.0 mmol/L range, which is tighter than other reports.
- SPRINT had very low hypoglycaemia (2% by patient) than other protocols with similar targets and, equally uniquely, TGC reduced hypoglycaemia by 50% from the retrospective comparator cohort.
- SPRINT provided more consistent control on a per-patient basis, in both tightness of control (variability) and target value (median glycaemia) than all other protocols who reported per-patient values. The middle 50% of SPRINT patients had median blood glucose within a 1.1 mmol/L wide band (5.5-6.6 mmol/L). Many TGC protocols do not report per patient results for both median and variability so comparison is difficult, but spread of reported patient means has been much larger, as well as a significant discussion point for several trials [51, 5, 52].
  - Figure A1-5 shows the median and IQR per-patient blood glucose distributions as cumulative distribution functions for both cohorts.
  - Figure A1-6 shows the cohort to cohort comparison of blood glucose
- SPRINT gave more insulin on a cohort and per-patient basis, but had less hypoglycaemia, contrary to many published studies and analyses [53].
- SPRINT gave less carbohydrate and total nutrition on average across the cohort, but the median SPRINT patient received more nutritional inputs (although lower carbohydrate loading) than in the Pre-SPRINT cohort (see Table 2 in [54]).
- Within this SPRINT cohort, there was no statistically significant association (P>0.3) between glycaemic outcome (average, range, maximum) and outcome. No other study reported this decoupling of outcome glycaemia and mortality.

Hence, SPRINT provided very tight control on a cohort and per-patient basis compared to all other published results (e.g. [43]), and succeeded in decoupling mortality and glycaemic outcome within the SPRINT cohort, which is also unique.


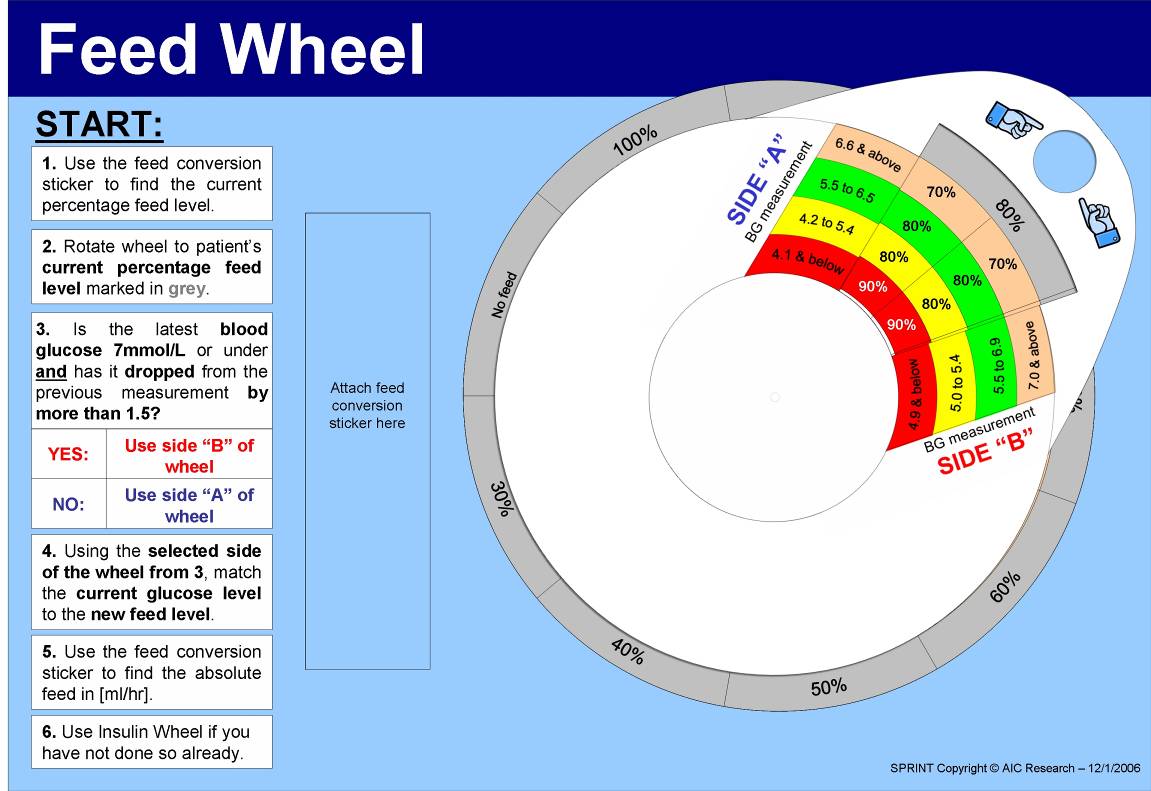


(a)


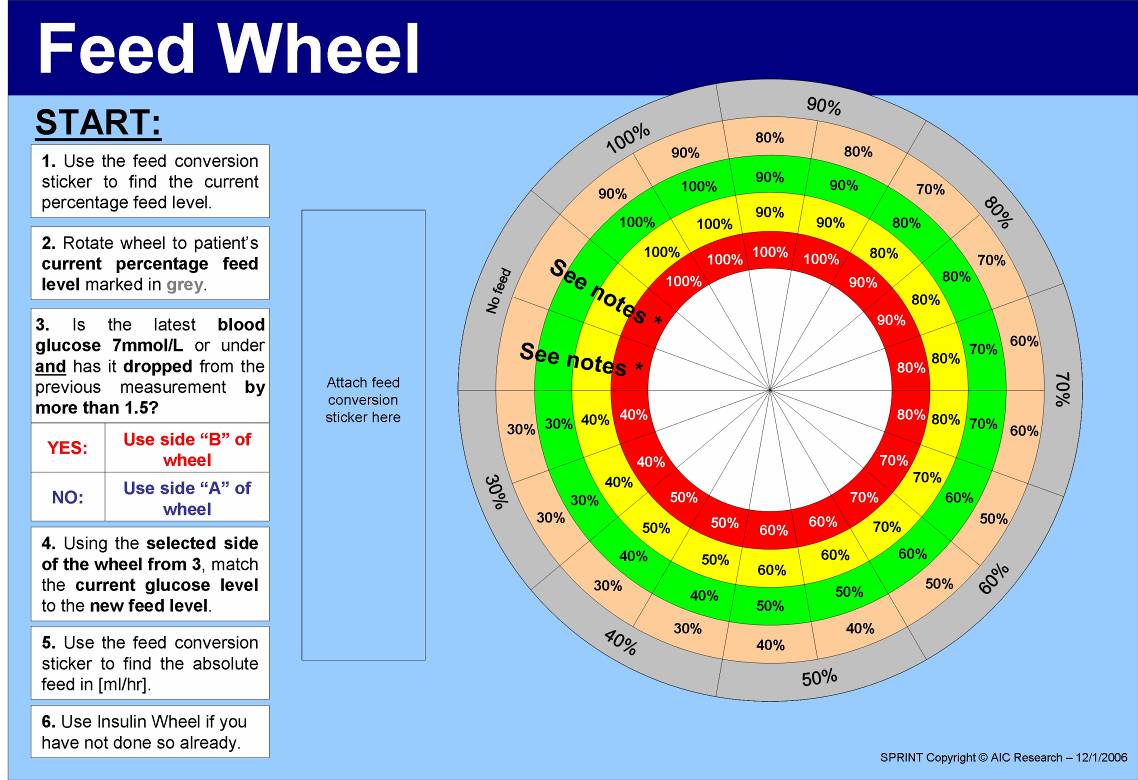


(b)

**Figure A1-1:** The SPRINT feed wheel with dial (a) and with dial removed (b). (Blood glucose values are in mmol/L, to convert to mg/dL multiply by 18).


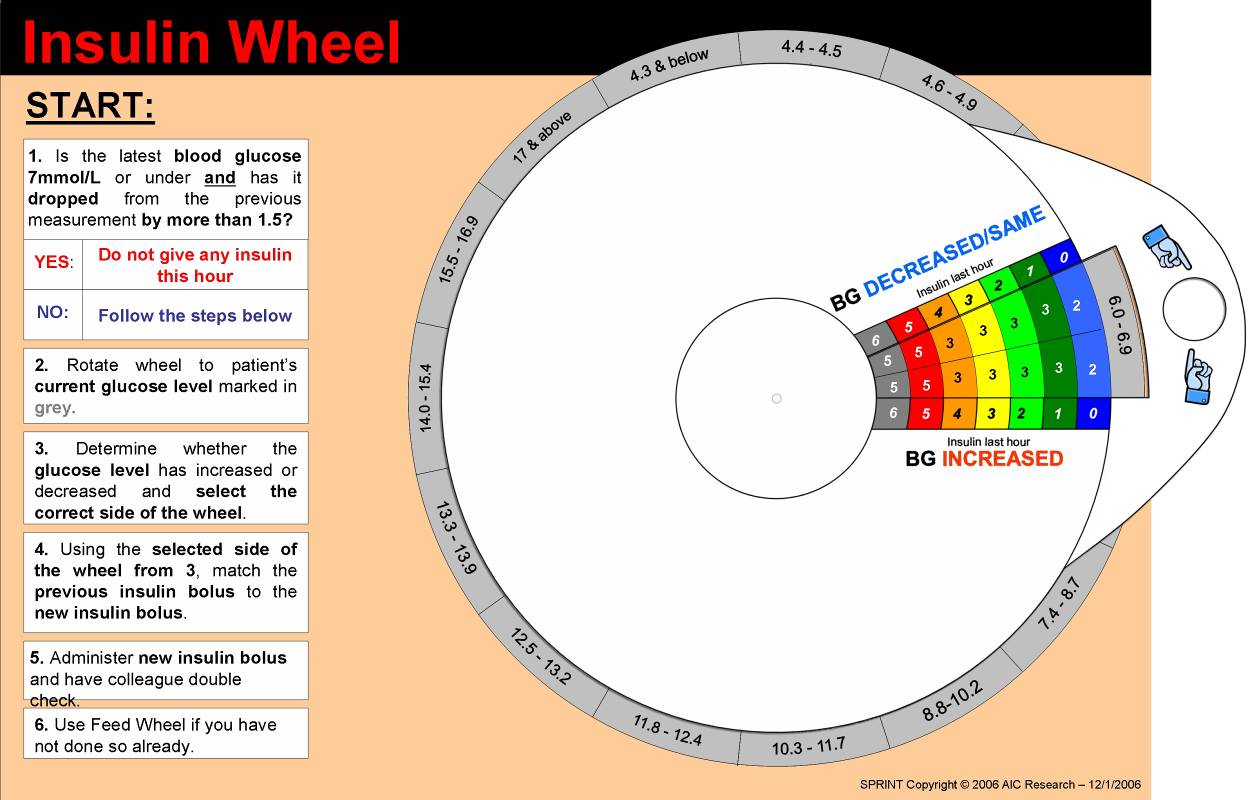


(a)


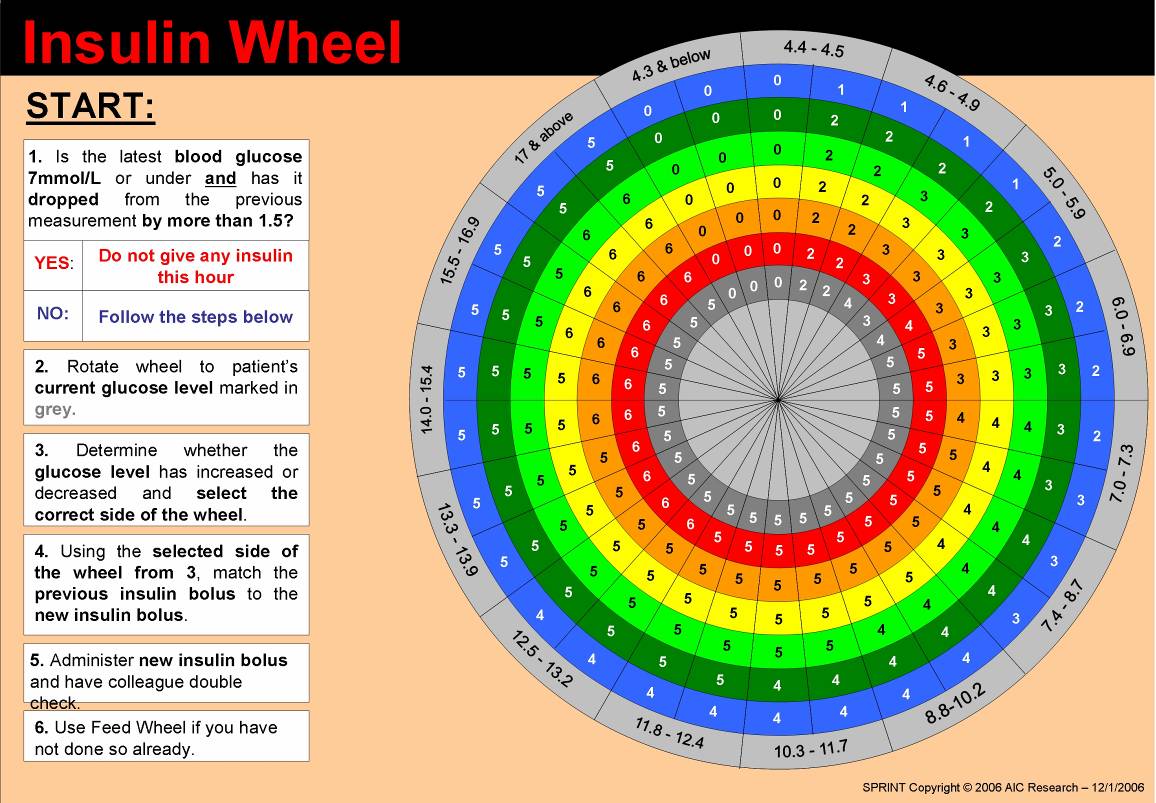


(b)

**Figure A1-2:** The SPRINT Insulin wheel with dial (a) and with dial removed (b). (Blood glucose values are in mmol/L, to convert to mg/dL multiply by 18).

**Figure A1-3:** Flow chart specifying guidelines for measuring blood glucose level two-hourly.

**Figure A1-4:** Flow chart specifying guidelines for stopping the SPRINT protocol


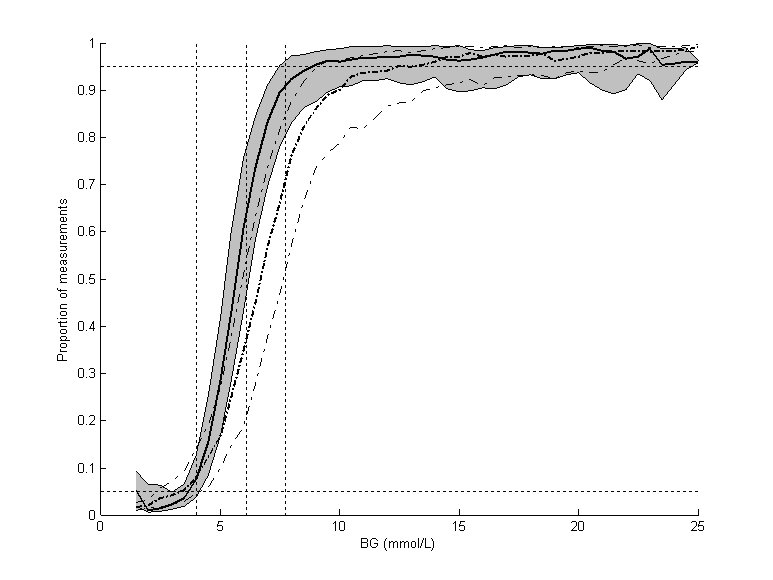


**Figure A1-5**: Per-patient cumulative distribution of blood glucose results for SPRINT and Pre-SPRINT. Solid lines represent the 25^th^, 50^th^ and 75^th^ percentiles for SPRINT patients. Dashed lines denote the same percentiles for the retrospective cohort.

SPRINT

Retrospective

**Figure A1-6**: Cohort wide cumulative distribution of blood glucose values for SPRINT and Pre-SPRINT cohorts.

**APPENDIX 1 REFERENCES**:

5. Finfer S, Chittock DR, Su SY, Blair D, Foster D, Dhingra V, Bellomo R, Cook D, Dodek P, Henderson WR *et al*: **Intensive versus conventional glucose control in critically ill patients**. *N Engl J Med* 2009, **360**(13):1283-1297.

14. Lonergan T, Le Compte A, Willacy M, Chase JG, Shaw GM, Hann CE, Lotz T, Lin J, Wong XW: **A pilot study of the SPRINT protocol for tight glycemic control in critically Ill patients**. *Diabetes Technol Ther* 2006, **8**(4):449-462.

20. Shaw GM, Chase JG, Wong J, Lin J, Lotz T, Le Compte AJ, Lonergan TR, Willacy MB, Hann CE: **Rethinking glycaemic control in critical illness - from concept to clinical practice change**. *Crit Care Resusc* 2006, **8**(2):90-99.

32. Goldberg PA, Siegel MD, Sherwin RS, Halickman JI, Lee M, Bailey VA, Lee SL, Dziura JD, Inzucchi SE: **Implementation of a safe and effective insulin infusion protocol in a medical intensive care unit**. *Diabetes Care* 2004, **27**(2):461-467.

41. Lonergan T, LeCompte A, Willacy M, Chase JG, Shaw GM, Wong XW, Lotz T, Lin J, Hann CE: **A simple insulin-nutrition protocol for tight glycemic control in critical illness: development and protocol comparison**. *Diabetes Technol Ther* 2006, **8**(2):191-206.

42. Krishnan JA, Parce PB, Martinez A, Diette GB, Brower RG: **Caloric intake in medical ICU patients: consistency of care with guidelines and relationship to clinical outcomes**. *Chest* 2003, **124**(1):297-305.

43. Suhaimi F, Le Compte A, Preiser JC, Shaw GM, Massion P, Radermecker R, Pretty CG, Lin J, Desaive T, Chase JG: **What makes tight glycemic control tight? The impact of variability and nutrition in two clinical studies**. *J Diabetes Sci Technol* 2010, **4**(2):284-298.

44. Natali A, Gastaldelli A, Camastra S, Sironi AM, Toschi E, Masoni A, Ferrannini E, Mari A: **Dose-response characteristics of insulin action on glucose metabolism: a non-steady-state approach**. *Am J Physiol Endocrinol Metab* 2000, **278**(5):E794-801.

45. Prigeon RL, Roder ME, Porte D, Jr., Kahn SE: **The effect of insulin dose on the measurement of insulin sensitivity by the minimal model technique. Evidence for saturable insulin transport in humans**. *J Clin Invest* 1996, **97**(2):501-507.

46. Chase JG, Shaw GM, Lin J, Doran CV, Hann C, Lotz T, Wake GC, Broughton B: **Targeted glycemic reduction in critical care using closed-loop control**. *Diabetes Technol Ther* 2005, **7**(2):274-282.

47. Chase JG, Shaw GM, Lin J, Doran CV, Bloomfield M, Wake GC, Broughton B, Hann C, Lotz T: **Impact of insulin-stimulated glucose removal saturation on dynamic modelling and control of hyperglycaemia**. *International Journal of Intelligent Systems Technologies and Applications (IJISTA)* 2004, **1**(1/2):79-94.

48. Rubinson L, Diette GB, Song X, Brower RG, Krishnan JA: **Low caloric intake is associated with nosocomial bloodstream infections in patients in the medical intensive care unit**. *Crit Care Med* 2004, **32**(2):350-357.

49. Cerra FB, Benitez MR, Blackburn GL, Irwin RS, Jeejeebhoy K, Katz DP, Pingleton SK, Pomposelli J, Rombeau JL, Shronts E *et al*: **Applied nutrition in ICU patients. A consensus statement of the American College of Chest Physicians**. *Chest* 1997, **111**(3):769-778.

50. Braithwaite SS, Edkins R, Macgregor KL, Sredzienski ES, Houston M, Zarzaur B, Rich PB, Benedetto B, Rutherford EJ: **Performance of a dose-defining insulin infusion protocol among trauma service intensive care unit admissions**. *Diabetes Technol Ther* 2006, **8**(4):476-488.

51. Preiser JC: **Year in review 2008: Critical Care--metabolism**. *Crit Care* 2009, **13**(5):228.

52. Mesotten D, Van den Berghe G: **Clinical benefits of tight glycaemic control: focus on the intensive care unit**. *Best Pract Res Clin Anaesthesiol* 2009, **23**(4):421-429.

53. Griesdale DE, de Souza RJ, van Dam RM, Heyland DK, Cook DJ, Malhotra A, Dhaliwal R, Henderson WR, Chittock DR, Finfer S *et al*: **Intensive insulin therapy and mortality among critically ill patients: a meta-analysis including NICE-SUGAR study data**. *Cmaj* 2009, **180**(8):821-827.

54. Lin J, Lee D, Chase JG, Shaw GM, Le Compte A, Lotz T, Wong J, Lonergan T, Hann CE: **Stochastic modelling of insulin sensitivity and adaptive glycemic control for critical care**. *Computer Methods and Programs in Biomedicine* 2008, **89**(2):141-152.
